# Editorial for the Special Issue on Gas Flows in Microsystems

**DOI:** 10.3390/mi10080494

**Published:** 2019-07-25

**Authors:** Stéphane Colin, Lucien Baldas

**Affiliations:** Institut Clément Ader (ICA), Université de Toulouse, CNRS-INSA-ISAE-Mines Albi-UPS, 31400 Toulouse, France

The last two decades have witnessed a rapid development of microelectromechanical systems (MEMS) involving gas microflows in various technical fields. Gas microflows can, for example, be observed in micro heat exchangers designed for chemical applications or for cooling of electronic components, in fluidic microactuators developed for active flow control purposes, in micronozzles used for the micropropulsion of nano- and picosatellites, in micro gas chromatographs, analyzers or separators, in vacuum generators and in Knudsen micropumps, as well as in some organs-on-a-chip such as artificial lungs. These flows are rarefied due to the small MEMS dimensions, and the rarefaction can be increased by low-pressure conditions. The flows relate to the slip flow, transition, or free molecular regimes, and can involve monatomic or polyatomic gases and gas mixtures. Hydrodynamics and heat and mass transfer are strongly impacted by rarefaction effects, and temperature-driven microflows offer new opportunities for designing original MEMS for gas pumping or separation. Accordingly, this Special Issue of *Micromachines*, entitled “Gas Flows in Microsystems” contains 14 papers (1 review and 13 research articles), which focus on novel theoretical and numerical models or data, as well as on new experimental results and techniques, for improving knowledge on heat and mass transfer in gas microflows. 

A few papers of this Special Issue have addressed fundamental issues on gas microflow modeling. Many microfluidic systems involving gases operate in the slip or early transition regimes, and the bulk flow can then be modeled in these slightly rarefied regimes by continuum approaches. In the Knudsen layer close to the walls, however, local thermodynamic disequilibrium takes place and specific approaches are required. An effective mean free path model was implemented by Bhagat et al. [1] in OpenFOAM, an open source computational fluid dynamics (CFD) code based on the Navier–Stokes equations. A hybrid Langmuir–Maxwell–Smoluchowski velocity slip and temperature jump boundary condition was used with a Knudsen layer formulation and tested on the backward facing step channel. Comparison with direct simulation Monte Carlo (DSMC) demonstrated a significant improvement over existing CFD solvers. Pressure drop in microchannels is a fundamental quantity to control for many engineering problems. In a number of devices, the entrance region is not negligible and should be taken into account. Duan et al. [2] proposed a semi-analytical model based on the momentum equation coupled with first-order slip boundary conditions. A good accuracy of this model, within 5%, was demonstrated in the slip flow regime by comparison with CFD simulations, as well as with experimental and numerical data from the literature. Even in non-rarefied regimes, the determination of friction factors is not straightforward, as demonstrated by Rehman et al. [3] who determined the average friction factor in gas flows along adiabatic microchannels with rectangular cross-section. From an experimental and numerical analysis, covering a large range of the Reynolds number from 200 to 20,000, they pointed out the role of minor loss coefficients and demonstrated that they should not be considered as constant. Gas microflows can also be encountered in gas microbearings where the aerodynamic lubrication performance has a critical impact on the stability of the bearing-rotor system in micromachines. The interactive effects of gas rarefaction and surface roughness on the static and dynamic characteristics of ultra-thin film gas lubrication in journal microbearings were investigated by Wu et al. [4] under various operative conditions and structure parameters. On the basis of the fractal geometry theory and the Boltzmann slip correction factor, the authors demonstrated that high values of the eccentricity ratio and bearing number tend to significantly increase the principal stiffness coefficients, and the fractal roughness surface considerably affects the ultra-thin film damping characteristics compared to smooth surface bearing. Controlling gas damping at microscale is also of high interest for the development of new compliant resonant microsystems. Mirzazadeh and Mariani [5] developed simple analytical solutions to estimate the dissipation in the ideal case of air flow between infinite plates, at atmospheric pressure, for application to comb-drive actuators. The results of numerical simulations were also reported to assess the effect of the finite size of actual geometries on damping.

These fundamental papers underline the importance of experimental data for validating simplified or more complex models. Unfortunately, the amount of experimental data on gas microflows is very limited, compared to the high number of numerical studies. The main difficulty, as explained in the review by Brandner [6], is due to the fact that conventional measurement techniques (for temperature, pressure, etc.) cannot be adapted to gas microflows, due to their intrusiveness and/or low signal delivery, especially when timely and spatially correlated measurements are required. In that review, the potential of nuclear magnetic resonance and magnetic resonance imaging for analyzing gas microflows is discussed. Some issues linked to the intrusiveness of sensors, even highly miniaturized, are also treated in the paper by Mironov et al. [7], in which the interaction between a Pitot microtube and a supersonic microjet is investigated.

The last series of papers published in this Special Issue are devoted to specific microsystems designed for the control or the analysis of gas microflows. One specific phenomenon experienced in rarefied gas flows is thermal transpiration, which allows the design of thermally driven pumps without any moving mechanical part. These so-called Knudsen pumps are very appealing for a number of applications requiring the control of a pressure, a flow rate, or the intake of a gas sample. Lopez Quesada et al. [8] provided some guidelines for the design of Knudsen micropumps based on architectures adapted to target applications which can require a high vacuum, a high flowrate, or a compromise between these two parameters. Their work is based on kinetic modeling and simulations, but takes into account some manufacturing constraints. Zhang et al. [9] focused their numerical analysis on the behavior of N_2_–O_2_ gas mixtures in a more classic design of the Knudsen pump. The thermal transpiration efficiency is related to the molecular mass of the gas and, even with a molecular mass close to that of O_2_, N_2_ was submitted to a stronger thermal transpiration effect. In addition, the lighter gas, N_2_, could effectively promote the motion of the heavier gas, O_2_. If separation of gas species from a mixture is of practical interest at a microfluidic level, it is also the case of mixing. Meskos et al. [10] numerically investigated the mixing process of two pressure-driven rarefied gas flows between parallel plates and evaluated the mixing length using a DSMC approach. They proposed a simple approach to control the output mixture composition, by only adding a splitter in an appropriate location of the microsystem’s mixing zone. This mixer was working in a steady state, differently from the option analyzed by Noël et al. [11] who proposed a new multi-stage design of pulsed micromixer. For example, they demonstrated that, for a 1 s pulse of pure gas (formaldehyde) followed by a 9 s pulse of pure carrier gas (air), an effective mixing up to 94–96% was obtained at the exit of the micromixer. There is currently a high demand for compact, accurate, and rapid gas detectors. Several papers in this Special Issue are focused on this subject. Khan et al. [12] developed a toluene detector based on deep ultraviolet (UV) absorption spectrophotometry. They implemented two types of hollow-core waveguides, namely, a glass capillary tube with aluminum-coated inner walls and an aluminum capillary tube, and obtained limits of detection of 8.1 ppm and 12.4 ppm, respectively. Rezende et al. [13] proposed a micro milled microfluidic photoionization detector of volatile organic compounds. The device does not require any glue, which facilitates the easy replacement of components, and the estimated detection limit is 0.6 ppm for toluene without any amplification unit. Finally, Lara-Ibeas et al. [14] developed a compact prototype of gas chromatograph equipped with a preconcentration unit, able to detect sub-ppb levels of benzene, toluene, ethylbenzene, and xylenes (BTEX) in gaseous mixtures. Detection limits of 0.20, 0.26, 0.49, 0.80, and 1.70 ppb were determined for benzene, toluene, ethylbenzene, m/p-xylenes, and o-xylene, respectively. 

We wish to thank all authors who submitted their papers to this Special Issue. We would also like to acknowledge all the reviewers for dedicating their time to provide careful and timely reviews to ensure the quality of this Special Issue.
